# Comparison of E,E-Farnesol Secretion and the Clinical Characteristics of *Candida albicans* Bloodstream Isolates from Different Multilocus Sequence Typing Clades

**DOI:** 10.1371/journal.pone.0148400

**Published:** 2016-02-05

**Authors:** Sook-In Jung, Jong Hee Shin, Soo Hyun Kim, Jin Kim, Joo Hee Kim, Min Ji Choi, Eun-Kyung Chung, Kyungwon Lee, Sun Hoe Koo, Hyun Ha Chang, Marie-Elisabeth Bougnoux, Christophe d’Enfert

**Affiliations:** 1 Department of Internal Medicine, Chonnam National University Medical School, Gwangju, South Korea; 2 Department of Laboratory Medicine, Chonnam National University Medical School, Gwangju, South Korea; 3 Biomedical Research Institute, Chonnam National University Hospital, Gwangju, Korea; 4 Department of Medical Education, Chonnam National University Medical School, Gwangju, South Korea; 5 Department of Laboratory Medicine and Research Institute of Bacterial Resistance, Yonsei University College of Medicine, Seoul, Korea; 6 Department of Laboratory Medicine, Chungnam National University College of Medicine, Daejeon, South Korea; 7 Department of Internal Medicine, Kyungpook National University School of Medicine, Daegu, South Korea; 8 Institut Pasteur, Unité Biologie et Pathogenicité Fongiques, F-75015 Paris, France; 9 INRA, USC2019, F-75015 Paris, France; 10 Laboratoire de Parasitologie-Mycologie, Service de Microbiologie, Hôpital Necker-Enfants Malades, Université Paris Descartes, Faculté de Médicine, F-75015 Paris, France; University of Birmingham, UNITED KINGDOM

## Abstract

Using multilocus sequence typing (MLST), *Candida albicans* can be subdivided into 18 different clades. Farnesol, a quorum-sensing molecule secreted by *C*. *albicans*, is thought to play an important role in the development of *C*. *albicans* biofilms and is also a virulence factor. This study evaluated whether *C*. *albicans* bloodstream infection (BSI) strains belonging to different MLST clades secrete different levels of E,E-farnesol (FOH) and whether they have different clinical characteristics. In total, 149 *C*. *albicans* BSI isolates from ten Korean hospitals belonging to clades 18 (n = 28), 4 (n = 23), 1 (n = 22), 12 (n = 17), and other clades (n = 59) were assessed. For each isolate, the FOH level in 24-hour biofilms was determined in filtered (0.45 μm) culture supernatant using high-performance liquid chromatography. Marked differences in FOH secretion from biofilms (0.10–6.99 μM) were observed among the 149 BSI isolates. Clade 18 isolates secreted significantly more FOH than did non-clade 18 isolates (mean ± SEM; 2.66 ± 0.22 *vs*. 1.69 ± 0.10 μM; *P* < 0.001). Patients with isolates belonging to clade 18 had a lower mean severity of illness than other patients, as measured using the “acute physiology and chronic health evaluation” (APACHE) III score (14.4 ± 1.1 *vs*. 18.0 ± 0.7; *P* < 0.05). This study provides evidence that *C*. *albicans* BSI isolates belonging to the most prevalent MLST clade (clade 18) in Korea are characterized by increased levels of FOH secretion and less severe illness.

## Introduction

*Candida albicans* is a commensal organism in healthy individuals, but it often causes nosocomial bloodstream infections (BSIs) [1]. Although BSI due to *C*. *albicans* is a major cause of morbidity and mortality in hospital patients worldwide [1,2], the pathogenesis of *C*. *albicans* BSI is complex and is not completely understood. *C*. *albicans* isolates can be assigned by multilocus sequence typing (MLST) to subsets of closely related strain types, referred to as clades [3,4]. To date, there are over 2,000 diploid sequence type (DST) profiles in the *C*. *albicans* MLST database from isolates recovered in multiple locations throughout the world, and *C*. *albicans* can be divided into 18 distinct clades (clades 1 to 18) [4,5]. Property differences among *C*. *albicans* clades have been described, exemplified for instance in the context of antifungal susceptibility, adhesion molecule structure, gene expression, or association with higher mortality of candidemia [3,6–8]. These previous reports suggest the possibility that clade-specific associations extend to properties of potential relevance to the role of *C*. *albicans* as a human commensal and pathogen [6,7].

Farnesol, a quorum-sensing molecule secreted by *C*. *albicans*, is considered to play an important role in the development of *C*. *albicans* biofilms [1,9–11]. Farnesol also plays a role in increasing the susceptibility of mice to systemic candidiasis, as well as decreasing the expression of T-cell and macrophage cytokines, indicating its role as a virulence factor [11]. Weber *et al*. [9] reported that *C*. *albicans* produces significant amounts of E,E-farnesol (FOH), compared with other *Candida* species, and FOH production differs markedly within *C*. *albicans* strains. Although *C*. *albicans* BSI is frequently associated with biofilm growth of *Candida* organisms on medical devices such as central venous catheters (CVC) [12–14], FOH production by BSI isolates of *C*. *albicans* and its genotype- or clade-specific differences have scarcely been assessed. In the current study, we aimed to evaluate whether *C*. *albicans* BSI strains belonging to different MLST clades or genotypes display different degrees of FOH secretion, and whether they exhibit different clinical characteristics.

## Materials and Methods

### *Candida albicans* BSI isolates and patient data

A total of 149 BSI isolates of *C*. *albicans* belonging to MLST clades 18 (n = 28), 4 (n = 23), 1 (n = 22), 12 (n = 17), and others (n = 59) were evaluated [5]. All isolates were obtained from blood cultures of 149 patients of 10 Korean university hospitals between September 2006 and August 2007. The 149 isolates yielded 108 DSTs: DST 727 belonging to clade 18 (12 isolates) was the most common MLST type, followed by 732 belonging to clade 18 (seven isolates), 69 belonging to clade 1 (six isolates), and 601 belonging to clade 12 (six isolates) (Table 1). Clinical information was collected retrospectively and included patient demographics, underlying diseases, clinical status at positive blood culture, antifungal therapy, and outcome of fungemia [15]. The Charlson comorbidity index was used to evaluate the patients’ comorbidities [16]. The severity of illness was assessed using the Acute Physiology and Chronic Health Evaluation III [APACHE III] score [17]. The APACHE III score is the sum of the acute physiology, age, and chronic health scores, ranging from 0 to 299, and is used to estimate the relative risk of hospital death [17]. To compare the clinical outcomes between groups, 14-day and 30-day mortalities after the initial diagnosis of candidemia were assessed. The outcome variable was defined as survival or death within 30 days of the first documented candidemia episode. This study was approved by the institutional review board of Chonnam National University Hospital (IRB CNUH-2014-290). A waiver of consent was granted given the retrospective nature of the project. The patient information was anonymized and de-identified prior to analysis, and no information was used that could lead to patient identification.

**Table 1:** E,E-farnesol secretion and biofilm formation by 149 bloodstream isolates of *Candida albicans* according to multilocus sequence typing (MLST) clade.

MLST		No. of isolates tested	Mean (SEM) of biofilm^b^		Mean (SEM) of E,E-farnesol^c^		
Clade	Diploid sequence type (DST)		XTT reduction (OD_492_)	Dry weight (mg)	Per culture (μM)	Per metabolic basis	Per weight basis
Clade 18	DST 727	12	0.297 (0.030)	2.78 (0.16)	2.90 (0.38)	10.91 (2.07)	1.04 (0.12)
	DST 732	7	0.261 (0.028)	2.46 (0.10)	2.52 (0.50)	11.24 (3.74)	1.07 (0.23)
	Other 9 DSTs	9	0.236 (0.039)	2.51 (0.29)	2.46 (0.28)	11.80 (1.53)	1.04 (0.13)
	Total	28	0.269 (0.019)	2.61 (0.12)	2.66 (0.22)	11.28 (1.32)	1.05 (0.08)
Clade 4	All 20 DSTs	23	0.261 (0.017)	2.50 (0.09)	1.29 (0.20)	5.34 (1.00)	0.53 (0.09)
Clade 1	DST 69	6	0.241 (0.032)	3.05 (0.48)	1.78 (0.36)	7.18 (1.05)	0.59 (0.09)
	Other 16 DSTs	16	0.303 (0.038)	2.72 (0.19)	2.04 (0.27)	8.87 (2.07)	0.79 (0.10)
	Total	22	0.286 (0.029)	2.81 (0.19)	1.97 (0.22)	8.41 (1.52)	0.74 (0.08)
Clade 12	DST 601	6	0.266 (0.038)	2.87 (0.24)	1.41 (0.52)	6.53 (2.88)	0.52 (0.20)
	Other 10 DSTs	11	0.247 (0.030)	2.86 (0.23)	1.89 (0.33)	7.73 (1.24)	0.68 (0.12)
	Total	17	0.254 (0.023)	2.86 (0.17)	1.72 (0.28)	7.30 (1.25)	0.63 (0.10)
Others^a^	DST 365	4	0.189 (0.056)	3.40 (0.39)	0.94 (0.25)	6.10 (2.02)	0.29 (0.10)
	Other 48 DSTs	55	0.264 (0.012)	2.71 (0.10)	1.78 (0.17)	7.56 (0.80)	0.67 (0.06)
	Total	59	0.259 (0.012)	2.76 (0.10)	1.73 (0.16)	7.46 (0.75)	0.65 (0.06)
Total, non-clade 18		121	0.263 (0.009)	2.73 (0.07)	1.69 (0.10) ^e^	7.21 (0.53)^e^	0.64 (0.04)^e^
Total, all isolates		149	0.264 (0.008)	2.71 (0.06)	1.87 (0.10)^d^	7.97 (0.51)^d^	0.72 (0.04)^d^

^a^ Includes clade 8 (12 isolates), clade 11 (5 isolates), clade 5 (5 isolates), clade 15 (5 isolates), clade 6 (4 isolates), clade 9 (4 isolates), clade 11b (4 isolates), clade 10 (2 isolates), 14 (1 isolate), 16 (1 isolate), and singletons (16 isolates).

^b^ Biofilm-forming abilities were determined by measuring XTT activity reduction and dry weight.

^c^ Quantification of E,E-farnesol from 149 bloodstream isolates of *C*. *albicans* was performed using high-performance liquid chromatography and FOH results (μM) obtained from each culture were also normalized on a metabolic basis or a per-weight basis by dividing the values by the biofilm results determined by XTT reduction (OD_492_) or dry weight (mg) assays, respectively.

^d^ *P* <0.05, significant difference of E,E-farnesol secretion among 5 different groups by one-way ANOVA.

^e^ *P* <0.05, significant difference of E,E-farnesol secretion between clade 18 and total non-clade 18 by independent sample t-test.

### Biofilm formation

Biofilm formation was assessed using a denture strip model and 12-well tissue culture plates, as described previously for *Candida* species [18,19]. Briefly, a standard inoculum of 10^7^ cells/ml from an overnight culture of the *C*. *albicans* strain was applied to the surface of a 1.5-cm^2^ denture strip (Nunc 174969 Thermanox Coverslips; diameter, 15 mm; Naperville, IL, USA), placed in a 12-well tissue culture plate with 4 ml phosphate-buffered saline (PBS). The cells were then allowed to adhere for 90 min at 37°C. Nonadherent cells were subsequently removed from the strips by gentle washing with PBS. The strips were then submerged in 4 ml yeast nitrogen base medium supplemented with 50 mM dextrose (YNBD) in 24-well tissue culture plates and incubated at 37°C for 24 h under continuous rotation at 125 rpm. Two milliliters of sterile-filtered (0.45 μm) culture supernatant was used for the determination of FOH [9], and the denture strips containing biofilm were used for the quantitation of biofilm formation [19]. Biofilm was quantified using both the colorimetric 2,3-bis (2-methoxy-4-nitro-5-sulfophenyl)-5((phenyl amino) carbonyl)-2H-tetrazolium hydroxide (XTT) assay and dry weight (DW) [19]. For XTT assay, strips containing biofilm were transferred to individual wells of a new 12-well plate containing 4 ml PBS/well. The plate was covered with aluminum foil after adding 50 μl XTT salt solution (1 mg/ml in PBS) and 4 μl menadione solution (1 mM in acetone; Sigma). The plates were incubated at 37°C for 5 h, after which the media were removed and centrifuged at 3,500 × *g* for 5 min at 4°C. XTT formazan in the supernatant was measured at 492 nm using a spectrophotometer [19]. For DW measurement, biofilms were scraped off the surface of the strips using a cell scraper, and both strips and scrapers were rinsed with PBS to remove residual biofilms. The material was filtered using a pre-weighed 0.22-μm-pore size filter under vacuum, dried in an incubator at 37°C for 48 h, and weighed (mg) [19].

### Determination of E,E-Farnesol

For quantification of FOH, 3 ml n-hexane/ethanol (9:1, v/v) were added to 1 ml sterile-filtered (0.45 μm) culture supernatant, and the derivatization of farnesol with 9-anthroylnitrile was performed as described previously [20]. The farnesol extracts were supplemented sequentially with 250 μl 0.08% 9-anthroylnitrile ethylacetate solution, 10 μl 0.2% 1-butanol ethylacetate solution as an internal standard, and 250 μl 0.2% quinuclidine ethylacetate solution. Sample analysis was performed using a Shim-Pack Vp-ODS column (5 μm, 150 × 4.6 mm; Shimadzu) equipped with a C_18_ guard cartridge (4.0 × 3.0 mm; Phenomenex) on a Shimadzu HPLC system (Kyoto, Japan). A mixture of acetonitrile and water (87:13, v/v) was used as the mobile phase at a flow rate of 1.5 ml/min. The farnesol derivative was detected using a fluorescence detector at an excitation wavelength of 363 nm and emission wavelength of 470 nm. Standard concentrations ranged from 0.1 to 2.0 μM. A weighted 1/concentration linear regression was used to obtain calibration curves from the standards. The regression equations of the calibration curves were used to calculate the concentrations of the samples. FOH results obtained from each culture were also normalized on a metabolic basis or a per-weight basis by dividing the values (μM) by the biofilm results determined by XTT reduction or DW assays, respectively.

### Statistical analysis

Statistical analysis was performed using SPSS version 20. Continuous variables were expressed as medians and ranges or means ± standard deviations. The independent t-test was used to compare continuous variables between two groups, the Mann-Whitney U test to compare continuous variables with a non-normal distribution, and one-way analysis of variance (ANOVA) with Tukey’s post hoc test to compare variables among more than three groups. Comparisons of categorical variables between groups were carried out using the χ^2^ or Fisher's exact t-test. The correlation between two continuous variables was assessed using Pearson’s test. To assess the relationship between 30-day mortality and a set of variables, a multiple logistic regression model was used. The results of these logistic regression analyses were reported as adjusted odds ratios (ORs) with 95% confidence intervals (CIs). *P* values < 0.05 were considered statistically significant.

## Results and Discussion

Recently, it has become clear that *C*. *albicans* BSIs are frequently associated with biofilm formation on CVCs [12–14, 21]. *C*. *albicans* is the third leading cause of catheter-related BSIs, with the second highest catheter colonization-to-infection rate [21]. Several reports have demonstrated that certain *C*. *albicans* BSI strains are more highly concentrated in particular geographic locales, and that established BSI strains are endemic in certain hospitals [22,23]. Also, our recent MLST study identified a new clade specific to Asia (clade 18) that contained the greatest proportion of BSI isolates from Korean hospitals (18.6%), followed by clades 4 (15.4%), 1 (14.7%), and 12 (11.5%) [5]. DST 727 and its single-locus variant (DST 732), which belong to clade 18, represented the most common MLST types among BSI isolates of *C*. *albicans* in Korea [5]. Because increasing use of CVC and the resultant biofilm is an important reason why the *C*. *albicans* BSI incidence continues to increase, we hypothesized that the prevalent clonal BSI strains of *C*. *albicans* possess higher biofilm-associated virulence traits to cause BSIs than do other isolates, or different clinical characteristics. In the current study, we investigated the secretion of FOH under biofilm conditions as well as the clinical characteristics of *C*. *albicans* BSI isolates, compared with those of known MLST types, which were recovered from 10 Korean hospitals over 1 year [5].

**Fig 1:**
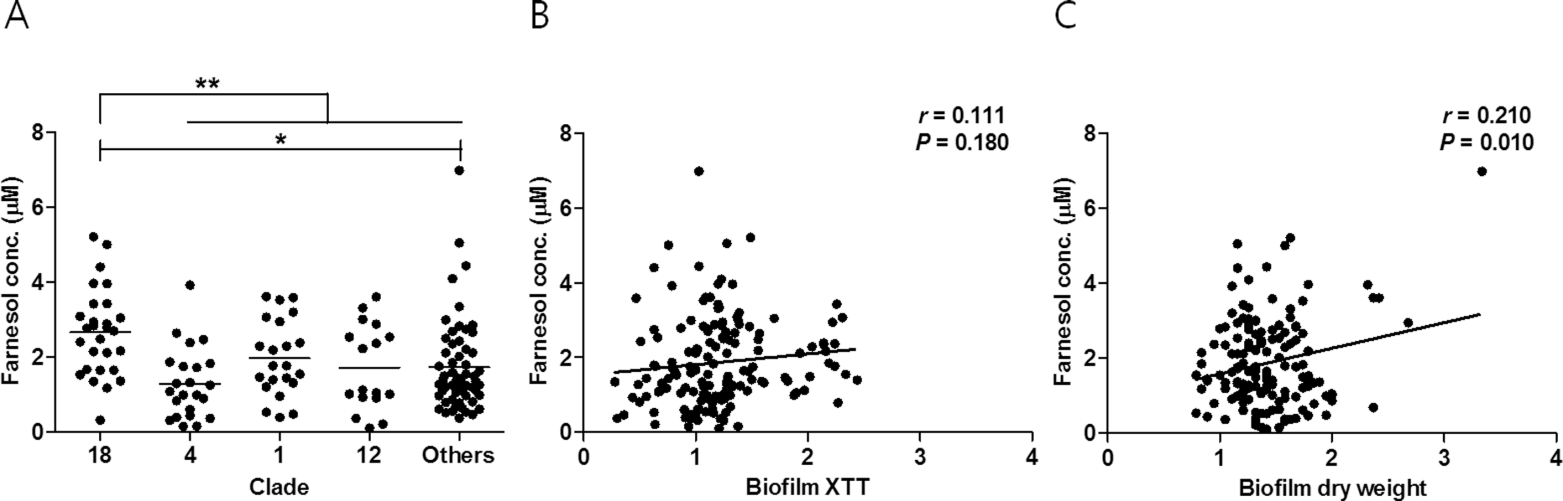
Secretion of E,E-farnesol (μM) of 149 bloodstream isolates of *Candida albicans* according to multilocus sequence typing clade (A) and their correlation of biofilm formation determined by measuring XTT activity reduction (B) and dry weight (C). Each symbol represents individual isolate. Horizontal lines indicate the mean farnesol values of the dataset at each clade. The biofilm results by both methods were normalized to those for *C*. *albicans* ATCC 90028 (set as 1.0). * Statistically significant (*p* < 0.05) compared among 5 different groups by one-way ANOVA. ** Statistically significant (*p* < 0.05) compared between clade 18 and total non-clade 18 by independent sample t-test.

The FOH secretion and biofilm formation by *C*. *albicans* BSI isolates of various MLST clades (clades 18, 4, 1, 12, and other clades) are presented in Table 1. All 149 *C*. *albicans* BSI isolates produced biofilms, and marked differences in FOH secretion (0.10–6.99 μM) were observed among the 149 BSI isolates under biofilm conditions (S1 Table), which confirms a previous report that individual *C*. *albicans* isolates varied remarkably in their ability to produce FOH [9]. The mean ± SEM levels of FOH (μM per culture basis) secreted by the isolates belonging to clades 18 (n = 28), 4 (n = 23), 1 (n = 22), 12 (n = 17), and others (n = 59) were 2.66 ± 0.22, 1.29 ± 0.20, 1.97 ± 0.22, 1.72 ± 0.28, and 1.73 ± 0.16, respectively; the differences among the five groups were found to be significant by one-way ANOVA with Tukey’s post hoc test (*P* = 0.001). *C*. *albicans* BSI isolates belonging to clade 18 showed significantly higher mean FOH secretion levels (2.66 ± 0.22 μM) than those of all other 121 isolates (1.69 ± 0.10 μM) (*P* < 0.001) (Fig 1A). The mean FOH secretion levels (μM) from isolates belonging to DST 727 (12 isolates) and DST 732 (7 isolates) were 2.90 ± 0.38 and 2.52 ± 0.50, respectively; both DSTs exhibited significantly higher FOH secretion levels than those of the other 130 BSI isolates (2.76 ± 0.30 *vs*. 1.74 ± 0.10 μM) (*P* < 0.001). The FOH values, which were normalized on a per weight basis (clade 18, 1.05 ± 0.08; non-clade 18, 0.64 ± 0.04, *P* < 0.001) or a metabolic basis (clade 18, 11.28 ± 1.32; non-clade 18, 7.21 ± 0.53, *P* = 0.002), also showed that clade 18 isolates produced greater levels of FOH. These results revealed that *C*. *albicans* BSI isolates belonging to the most prevalent MLST clade (clade 18) or the most prevalent DST types (DSTs 727 and 732) in Korea showed higher FOH production under biofilm conditions.

The mean biofilm-forming abilities of *C*. *albicans* BSI isolates determined by both XTT reduction and DW assays were similar among isolates from different MLST clades (both, *P >* 0.05) (Table 1). The levels of FOH secretion by *C*. *albicans* showed a slight correlation with the amount of biofilm formation determined by DW (*r* = 0.210, *P* = 0.010) (Fig 1B), while it did not show a significant correlation with the amount of biofilm formation by the XTT method (*r* = 0.111, *P* = 0.180) (Fig 1C), which is consistent with a previous report [9,24]. There was no correlation between the amount of biofilm determined by the XTT method and that by the DW method (*r* = -0.146, *P* = 0.075). The reasons for these discrepant results are unclear. One possible explanation is that some strains that produce abundant biofilm may be shifting metabolism away from routine functions, while strains producing low levels of biofilm or no biofilm might in fact be expected to show a higher level of metabolic activity as determined by XTT assay [25]. In addition, farnesol can be produced under biofilm conditions, but it can inhibit biofilm formation [26]. If the interior of biofilms is anaerobic, then larger biofilms should produce less farnesol, since anaerobic *C*. *albicans* does not produce farnesol [27] and thus farnesol production may be correlated with the surface area of the biofilm, rather than the total mass.

Table 2 lists the clinical characteristics of the 149 patients with candidemia according to clade. There were no significant differences among patients harboring different *C*. *albicans* clades with respect to age, sex, or underlying diseases. However, some findings showed the possibility of clade-specific or genotype-specific differences in clinical characteristics among *C*. *albicans* BSI isolates. Patients receiving total parenteral nutrition were infected more commonly by clade 12 than all non-clade 12 (132 isolates) groups (82.4% vs. 47.7%, *P* = 0.007). Patients chronic lung disease were infected more commonly by clade 1 than all non-clade 1 (127 isolates) groups (31.8% vs. 12.6%, *P* = 0.048). Notably, the APACHE III score was lower in patients infected with clade 18 than all non-clade 18 (130 isolates) groups (14.4 ± 1.1 *vs*. 18.0 ± 0.7, *P* = 0.024). In addition, patients infected with *C*. *albicans* BSI isolates belonging to DST 727 and DST 732 showed lower APACHE III scores than those of patients infected with *C*. *albicans* BSI isolates belonging to other DSTs (13.8 ± 1.3 vs. 17.8 ± 0.7, *P* = 0.033). These results show that patients with clade 18 strains had a lower mean severity of illness than that of other patients, as measured by the APACHE III score.

**Table 2:** Clinical characteristics of *Candida albicans* bloodstream isolates from different MLST clades.

Characteristics ^a^	Clade 18 (N = 28)	Non-clade 18 (N = 121)				
		Clade 4 (N = 23)	Clade 1 (N = 22)	Clade 12 (N = 17)	Others (N = 59)	Total (N = 121)
Demographic characteristics						
Age, years, mean ± SEM	63.6 ± 3.9	64.6 ± 2.9	59.9 ± 4.8	67.4 ± 3.1	54.9 ± 3.1	59.4 ± 1.9
Age ≤48 years, no. (%)	3 (10.7)	3 (13.0)	4 (18.2)	1 (5.9)	13 (22.0)	21 (17.4)
Male sex, no. (%)	21 (75.0)	14 (60.9)	12 (54.5)	14 (82.4)	29 (49.2)	69 (57.0)
Underlying disease, no. (%)						
Malignant tumor	11 (39.3)	8 (34.8)	11 (50.0)	6 (35.3)	22 (37.3)	47 (38.8)
Diabetes mellitus	6 (21.4)	9 (39.1)	4 (18.2)	5 (29.4)	11 (18.6)	29 (24.0)
Cerebrovascular disease	2 (7.1)	3 (13.0)	4 (18.2)	4 (23.5)	11 (18.6)	22 (18.2)
Chronic lung disease	4 (14.3)	4 (17.4)	7 (31.8)^b^	3 (17.6)	5 (8.5)	19 (15.7)
Moderate or severe kidney disease	1 (3.6)	2 (8.7)	2 (9.1)	2 (11.8)	13 (22.0)	19 (15.7)
Charlson comorbidity index, mean ± SD	6.6 ± 6.1	6.3 ± 5.4	6.1 ± 5.4	7.1 ± 6.1	5.8 ± 5.2	6.2 ± 5.3
Clinical status at positive blood culture, no. (%)						
Neutropenia	2 (7.1)	3 (13.0)	2 (9.1)	0 (0.0)	3 (5.1)	8 (6.6)
Immunosuppressive therapy	4 (14.3)	2 (8.7)	2 (9.1)	1 (5.9)	3 (5.1)	8 (6.6)
Total parenteral nutrition	14 (50.0)	9 (39.1)	12 (54.5)	14 (82.4)^b^	28 (48.3)	63 (52.5)
Surgery within 30 days	10 (35.7)	8 (34.8)	8 (36.4)	5 (29.4)	13 (22.0)	34 (28.1)
ICU admission at positive culture	10 (35.7)	9 (39.1)	9 (40.9)	6 (35.3)	22 (37.3)	46 (38.0)
Previous use of antifungal agent	2 (7.1)	3 (13.0)	2 (9.1)	0 (0.0)	5 (8.5)	10 (8.3)
Concomittant bacateremia	5 (17.9)	9 (39.1)	2 (9.1)	4 (23.5)	15 (25.4)	30 (24.8)
Indwelling urinary catheter	17 (60.7)	13 (56.5)	14 (63.6)	12 (70.6)	37 (62.7)	76 (62.8)
Presence of CVC	21 (75.0)	17 (73.9)	17 (77.3)	11 (64.7)	39 (66.1)	84 (69.4)
CVC-related candidmia	10 (35.7)	11 (47.8)	9 (40.9)	7 (41.2)	16 (27.1)	43 (35.5)
APACHE III score, mean ± SEM	14.4 ± 1.1	18.0 ± 1.7	18.1 ± 1.3	16.9 ± 1.8	18.3 ± 1.1	18.0 ± 0.7^c^
Therapy, no. (%)						
Catheter non-removal	5/21 (23.8)	2/17 (11.8)	6/17 (35.3)	4/11 (36.4)	8/39 (20.5)	20/84 (23.8)
Antifungal therapy after diagnosis	20 (71.4)	19 (82.6)	16 (72.7)	9 (52.9)	46 (78.0)	90 (74.4)
Outcomes, no. with indicated result / total no. (%)						
14-day mortality	8/28 (28.6)	9/23 (39.1)	8/21 (38.1)	8/16 (50.0)	15/54 (27.8)	40/114 (35.1)
30-day mortality	9/26 (34.6)	11/23 (47.8)	9/20 (45.0)	9/16 (56.2)	22/52 (42.3)	51/111 (45.9)

^a^ CVC, central venous catheter; ICU, intensive care unit; APACHE III score, “acute physiology and chronic health evaluation” III score.

^b^ *P* <0.05, significant difference between a given clade and the others (clade 12 vs. all non-clade 12; clade 1 vs. all non clade 1) by χ^2^ or Fisher's exact t-test.

^c^*P* <0.05, significant difference in APACHE III score between clade 18 and all non-clade 18 by independent sample t-test.

In the current study, >70% (105/149) of all patients were using CVC at the time of positive blood cultures, and only 35.6% (53/149) were diagnosed as having CVC-related fungemia (Table 2); these findings are consistent with previous reports [14,28]. The overall 30-day mortality rate for *C*. *albicans* candidemia was 43.7% (60/137), which is consistent with a recent report [29]. The lowest 30-day mortality rate was 34.6% for patients with clade 18 isolates, followed by 45.0% with clade 1 isolates, 47.8% with clade 4 isolates, and 56.2% with clade 12 isolates, but the differences were not statistically significant (*P* = 0.714). Initially, we supposed that *C*. *albicans* strains of the clade 18 genotype may be associated with a different frequency of CVC-related candidemia or different mortality rate than other strains because of their higher production of FOH under biofilm conditions. However, we did not find any clade-specific differences in the presence of CVC (clade 18, 75.0%; non-clade 18, 69.4%), CVC-related candidemia (clade 18, 35.7%; non-clade 18, 35.5%), or overall 30-day mortality rate (clade 18, 34.6%; non-clade 18, 45.9%).

Table 3 shows the multiple logistic regression analysis performed to identify risk factors for 30-day mortality. Neither the MLST clade nor FOH concentration showed an association with 30-day mortality, suggesting that higher levels of farnesol were not responsible for clinical outcome. Only two factors, the APACHE III score and surgery within 30 days prior to candidemia, showed an association with 30-day mortality. The APACHE III score increased 30-day mortality (OR, 1.073, 95% CI, 1.014–1.137, *P* = 0.015), suggesting it to be a predictive factor for 30-day mortality, which is also consistent with previous reports [29,30]. In addition, surgery within 30 days prior to candidemia decreased 30-day mortality (OR, 0.360, 95% CI, 0.151–0.858, *P* = 0.021). Several previous reports [1,31] have also shown that candidemic patients who had undergone prior surgery showed better outcomes than did those who had not, because the source of candidemia caused by the surgically induced violation of the gastrointestinal mucosa might be easily controlled by appropriate management, in contrast with candidemic patients with underlying medical conditions such as cirrhosis, neutropenia, or steroid use [31].

**Table 3:** Predictive factors for 30-day mortality by multiple logistic regression analysis^a^.

Variable	Category	Adjusted OR (95% CI)	*P* value
Gender	Male	1.537 (0.686–3.445)	0.296
	Female	1.000	
Age (year)		1.016 (0.996–1.036)	0.112
Clade	Non-clade 18	1.000	0.409
	Clade 18	0.648 (0.231–1.815)	
Surgery within 30 days prior to candidemia	No	1.000	0.021
	Yes	0.360 (0.151–0.858)	
ICU admission at positive culture (ref = No)	No	1.000	0.329
	Yes	1.593 (0.625–4.056)	
Indwelling urinary catheter (ref = No)	No	1.000	0.411
	Yes	1.427 (0.612–3.330)	
APACHE III		1.073 (1.014–1.137)	0.015
E,E-farnesol secretion (μM)		1.050 (0.751–1.470)	0.774

^a^ OR, odds ratio; CI, confidence interval; ICU, intensive care unit.

To date, no substantial study has compared various clinical characteristics among different clades of *C*. *albicans* BSI isolates, except for one recent study [8]. That report showed that BSI isolates belonging to the “general-purpose genotype” (GPG; corresponding to clade 1) are associated with increased mortality in patients with candidemia, albeit only in younger (<48 years) patients [8]. In the current study, only 16.1% (24/149) of the total patients were of young age (<48 years), and we did not find any clade-specific differences in age or mortality among the patients. Clade 1 accounted for the largest proportion of *C*. *albicans* isolates [3,6], but it contained a lower proportion of blood isolates than commensal isolates [3]. Our MLST study of oral commensal *C*. *albicans* isolates from healthy individuals in Korea found that clade 1 comprised the greatest proportion of isolates (21.6%), followed by clades 12 (18.9%), 11 (13.5%), 4 (10.8%), and 18 (5.4%) (data not shown), suggesting that in comparison, clade 18 was particularly enriched in BSI isolates. When only clade 18 and clade 1 strains were considered in the current study, the patients with clade 1 showed significantly higher mean APACHE III scores than those of patients with clade 18 strains (*P = 0*.*032*), and they tended to be associated with higher 30-day mortality rates (clade 1, 45.0%; clade 18, 34.6%, *P = 0*.*550*), suggesting that clade 1 strains may possess certain virulence factors associated with severe illness or mortality, which may be different from those of clade 18 strains. Several comparative studies between clade 1 strains and other clade strains have identified several virulence determinants, such as increased resilience to chemicals, increased adhesion, GPG-specific alleles of DNA tandem repeat-containing genes, and genes involved in dimorphism [8].

High farnesol-producing biofilms may correlate with the tendency of that biofilm to shed free yeast cells [26], which may or may not correlate with more severe clinical symptoms. In the present study, clade 18 BSI isolates showed higher FOH production, and the patients with clade 18 strains showed a lower mean severity of illness than that of other patients, as measured by the APACHE III score. However, FOH secretion was not correlated with a decreased APACHE III score (*r* = –0.136, *P* = 0.099), and similar results were obtained from the FOH values, which were normalized on a per weight basis or a metabolic basis. These results suggest that higher FOH levels may not to predict less severe illness, and that increased FOH secretion and the propensity to cause less severe illness are two independent properties of clade 18 strains.

The pathologic role of FOH remains an open question. Initially, it was thought that farnesol could be manipulated to treat invasive candidiasis as a fungistatic agent [1,32], but the discovery that endogenous farnesol actually contributed to *C*. *albicans* virulence has redirected recent research toward understanding quorum-sensing molecules as an important virulence factor of systemic candidiasis [1,11,33]. A recent study showed that secretion of farnesol may promote *C*. *albicans* dissemination through macrophages which have an important role in early detection and elimination of *C*. *albicans* in the host [34]. Considering the report that FOH may play an important virulence role in destruction of the host epithelial cell layer as the initial invasion process [24], farnesol may act as a virulence factor before, rather than after, *C*. *albicans* reaches the bloodstream. Biofilm formation is associated with an enhanced capacity of *C*. *albicans* to colonize indwelling CVCs, thus providing a reservoir from which the organism may enter the bloodstream [21]. Therefore, FOH produced under biofilm conditions, as a virulence factor, may be involved in the initial BSI invasion process. In the present study, we showed for the first time that clade 18 isolates produced a greater quantity of FOH under biofilm conditions, as compared with non-clade 18 isolates, in candidemic patients. The high prevalence of clade 18 strains as BSI pathogens in Korea suggests that specific microbial factors, including FOH, secreted under biofilm conditions could contribute to the increased ability of *C*. *albicans* clade 18 strains to cause BSIs, which have an impact on the epidemiology of *C*. *albicans* BSIs in Korea.

## Supporting Information

S1 Table The results of mutilocus sequence typing (MLST), biofilm formation, and farnesol secretion for all 149 bloodstream isolates of *C*. *albicans*. (DOCX)
