# Supplementary material for: Comparison of E,E-Farnesol Secretion and the Clinical Characteristics of Candida albicans Bloodstream Isolates from Different Multilocus Sequence Typing Clades
Source: PLoS One. 2016 Feb 5;11(2):e0148400. doi: 10.1371/journal.pone.0148400 (PMC4743943; doi:10.1371/journal.pone.0148400)
Supplement: S1 Table — (DOCX) [file pone.0148400.s001.docx]

**Supporting Information**

| Table S1. The results of mutilocus sequence typing (MLST), biofilm formation, and farnesol secretion for all 149 bloodstream isolates of *C. albicans* | | | | | | | |
| --- | --- | --- | --- | --- | --- | --- | --- |
| Isolate No. | MLST | | Biofilm | | Farnesol | | |
|  | Clade | DST | XTT reduction (OD_492_) | Dry weight (mg) | Per culture (μM)^a^ | Per metabolic basis^b^ | Per dry weight basis^c^ |
| 1 | 18 | 727 | 0.247 | 2.4 | 2.40 | 9.71 | 1.00 |
| 2 | 18 | 727 | 0.165 | 3.0 | 5.01 | 30.39 | 1.67 |
| 3 | 18 | 727 | 0.470 | 2.7 | 2.17 | 4.61 | 0.80 |
| 4 | 18 | 727 | 0.161 | 1.6 | 1.18 | 7.31 | 0.74 |
| 5 | 18 | 727 | 0.324 | 3.1 | 5.22 | 16.12 | 1.68 |
| 6 | 18 | 727 | 0.299 | 3.1 | 3.09 | 10.34 | 1.00 |
| 7 | 18 | 727 | 0.492 | 2.3 | 3.43 | 6.97 | 1.49 |
| 8 | 18 | 727 | 0.219 | 2.5 | 1.67 | 7.64 | 0.67 |
| 9 | 18 | 727 | 0.347 | 3.6 | 1.36 | 3.92 | 0.38 |
| 10 | 18 | 727 | 0.243 | 2.4 | 2.77 | 11.41 | 1.16 |
| 11 | 18 | 727 | 0.262 | 3.4 | 3.97 | 15.14 | 1.17 |
| 12 | 18 | 727 | 0.339 | 3.2 | 2.48 | 7.31 | 0.77 |
| 13 | 18 | 732 | 0.261 | 2.3 | 3.42 | 13.09 | 1.49 |
| 14 | 18 | 732 | 0.138 | 2.2 | 4.41 | 31.95 | 2.00 |
| 15 | 18 | 732 | 0.279 | 2.3 | 2.12 | 7.60 | 0.92 |
| 16 | 18 | 732 | 0.371 | 2.4 | 3.05 | 8.22 | 1.27 |
| 17 | 18 | 732 | 0.223 | 3.0 | 0.32 | 1.42 | 0.11 |
| 18 | 18 | 732 | 0.312 | 2.4 | 1.65 | 5.30 | 0.69 |
| 19 | 18 | 732 | 0.243 | 2.6 | 2.70 | 11.10 | 1.04 |
| 20 | 18 | 463 | 0.062 | 2.0 | 1.35 | 21.74 | 0.67 |
| 21 | 18 | 1513 | 0.234 | 2.0 | 2.84 | 12.16 | 1.42 |
| 22 | 18 | 1596 | 0.181 | 3.4 | 1.53 | 8.46 | 0.45 |
| 23 | 18 | 1597 | 0.205 | 1.6 | 2.15 | 10.48 | 1.34 |
| 24 | 18 | 1598 | 0.29 | 4.4 | 3.96 | 13.65 | 0.90 |
| 25 | 18 | 1599 | 0.304 | 2.4 | 2.89 | 9.51 | 1.20 |
| 26 | 18 | 1600 | 0.189 | 1.9 | 2.79 | 14.76 | 1.47 |
| 27 | 18 | 1601 | 0.484 | 2.2 | 2.94 | 6.07 | 1.33 |
| 28 | 18 | 1602 | 0.176 | 2.7 | 1.65 | 9.38 | 0.61 |
| 29 | 4 | 659 | 0.145 | 2.6 | 1.08 | 7.46 | 0.42 |
| 30 | 4 | 659 | 0.409 | 2.2 | 0.99 | 2.43 | 0.45 |
| 31 | 4 | 462 | 0.172 | 2.2 | 1.72 | 9.99 | 0.78 |
| 32 | 4 | 623 | 0.285 | 3.7 | 0.99 | 3.49 | 0.27 |
| 33 | 4 | 915 | 0.172 | 2.3 | 1.87 | 10.89 | 0.81 |
| 34 | 4 | 1539 | 0.172 | 2.1 | 3.92 | 22.78 | 1.87 |
| 35 | 4 | 1547 | 0.275 | 2.4 | 0.90 | 3.27 | 0.37 |
| 36 | 4 | 1571 | 0.208 | 2.0 | 0.36 | 1.73 | 0.18 |
| 37 | 4 | 1573 | 0.273 | 2.5 | 0.31 | 1.13 | 0.12 |
| 38 | 4 | 1574 | 0.28 | 2.8 | 1.30 | 4.64 | 0.46 |
| 39 | 4 | 1605 | 0.343 | 2.5 | 2.64 | 7.69 | 1.05 |
| 40 | 4 | 1606 | 0.196 | 2.3 | 0.60 | 3.05 | 0.26 |
| 41 | 4 | 1607 | 0.252 | 3.3 | 1.83 | 7.27 | 0.56 |
| 42 | 4 | 1608 | 0.284 | 2.6 | 2.47 | 8.71 | 0.95 |
| 43 | 4 | 1608 | 0.229 | 2.9 | 0.84 | 3.65 | 0.29 |
| 44 | 4 | 1609 | 0.198 | 2.5 | 0.39 | 1.95 | 0.15 |
| 45 | 4 | 1609 | 0.204 | 2.9 | 0.15 | 0.73 | 0.05 |
| 46 | 4 | 1610 | 0.329 | 2.2 | 1.75 | 5.33 | 0.80 |
| 47 | 4 | 1611 | 0.467 | 2.6 | 2.39 | 5.13 | 0.92 |
| 48 | 4 | 1612 | 0.3 | 2.6 | 0.16 | 0.52 | 0.06 |
| 49 | 4 | 1613 | 0.217 | 1.7 | 0.44 | 2.04 | 0.26 |
| 50 | 4 | 1629 | 0.352 | 2.2 | 1.32 | 3.76 | 0.60 |
| 51 | 4 | 1630 | 0.245 | 2.5 | 1.25 | 5.11 | 0.50 |
| 52 | 1 | 69 | 0.341 | 3.4 | 2.19 | 6.43 | 0.65 |
| 53 | 1 | 69 | 0.166 | 1.7 | 1.44 | 8.66 | 0.85 |
| 54 | 1 | 69 | 0.236 | 2.4 | 1.20 | 5.09 | 0.50 |
| 55 | 1 | 69 | 0.138 | 2.6 | 0.53 | 3.87 | 0.21 |
| 56 | 1 | 69 | 0.294 | 3.1 | 2.38 | 8.09 | 0.77 |
| 57 | 1 | 69 | 0.27 | 5.1 | 2.95 | 10.92 | 0.58 |
| 58 | 1 | 24 | 0.302 | 2.1 | 3.20 | 10.59 | 1.52 |
| 59 | 1 | 37 | 0.102 | 2.8 | 3.59 | 35.18 | 1.28 |
| 60 | 1 | 59 | 0.14 | 3.2 | 1.77 | 12.65 | 0.55 |
| 61 | 1 | 66 | 0.503 | 2.3 | 3.07 | 6.10 | 1.33 |
| 62 | 1 | 185 | 0.511 | 1.5 | 1.55 | 3.04 | 1.03 |
| 63 | 1 | 254 | 0.206 | 3.2 | 0.39 | 1.88 | 0.12 |
| 64 | 1 | 444 | 0.247 | 4.5 | 3.62 | 14.64 | 0.80 |
| 65 | 1 | 766 | 0.233 | 3.3 | 3.53 | 15.14 | 1.07 |
| 66 | 1 | 1097 | 0.488 | 2.5 | 1.77 | 3.63 | 0.71 |
| 67 | 1 | 1555 | 0.153 | 2.3 | 1.31 | 8.57 | 0.57 |
| 68 | 1 | 1556 | 0.533 | 1.7 | 1.40 | 2.63 | 0.82 |
| 69 | 1 | 1562 | 0.393 | 2.7 | 1.46 | 3.71 | 0.54 |
| 70 | 1 | 1565 | 0.257 | 3.0 | 2.29 | 8.93 | 0.76 |
| 71 | 1 | 1566 | 0.209 | 3.7 | 0.48 | 2.30 | 0.13 |
| 72 | 1 | 1567 | 0.445 | 2.4 | 2.29 | 5.15 | 0.96 |
| 73 | 1 | 1568 | 0.123 | 2.3 | 0.96 | 7.80 | 0.42 |
| 74 | 12 | 601 | 0.430 | 2.3 | 1.12 | 2.61 | 0.49 |
| 75 | 12 | 601 | 0.143 | 2.2 | 2.54 | 17.75 | 1.15 |
| 76 | 12 | 601 | 0.233 | 3.8 | 1.02 | 4.36 | 0.27 |
| 77 | 12 | 601 | 0.261 | 3.1 | 3.32 | 12.71 | 1.07 |
| 78 | 12 | 601 | 0.264 | 2.7 | 0.10 | 0.38 | 0.04 |
| 79 | 12 | 601 | 0.266 | 3.1 | 0.36 | 1.36 | 0.12 |
| 80 | 12 | 299 | 0.14 | 2.5 | 0.21 | 1.53 | 0.09 |
| 81 | 12 | 369 | 0.301 | 2.5 | 3.01 | 10.00 | 1.20 |
| 82 | 12 | 719 | 0.273 | 2.8 | 1.01 | 3.70 | 0.36 |
| 83 | 12 | 719 | 0.097 | 3.0 | 0.93 | 9.63 | 0.31 |
| 84 | 12 | 1546 | 0.489 | 1.8 | 2.37 | 4.84 | 1.32 |
| 85 | 12 | 1551 | 0.239 | 2.1 | 0.91 | 3.80 | 0.43 |
| 86 | 12 | 1557 | 0.256 | 2.9 | 2.88 | 11.26 | 0.99 |
| 87 | 12 | 1572 | 0.221 | 2.5 | 2.23 | 10.08 | 0.89 |
| 88 | 12 | 1615 | 0.237 | 4.6 | 3.61 | 15.22 | 0.78 |
| 89 | 12 | 1616 | 0.19 | 3.8 | 1.05 | 5.51 | 0.28 |
| 90 | 12 | 1619 | 0.27 | 2.9 | 2.54 | 9.41 | 0.88 |
| 91 | 8 | 365 | 0.282 | 3.4 | 1.26 | 4.45 | 0.37 |
| 92 | 8 | 365 | 0.285 | 4.5 | 0.68 | 2.40 | 0.15 |
| 93 | 8 | 365 | 0.122 | 2.7 | 1.44 | 11.79 | 0.53 |
| 94 | 8 | 365 | 0.065 | 3.0 | 0.37 | 5.74 | 0.12 |
| 95 | 8 | 1512 | 0.241 | 2.9 | 2.02 | 8.40 | 0.70 |
| 96 | 8 | 1512 | 0.328 | 2.8 | 1.41 | 4.31 | 0.50 |
| 97 | 8 | 1621 | 0.396 | 2.2 | 1.37 | 3.46 | 0.62 |
| 98 | 8 | 1622 | 0.321 | 2.1 | 1.63 | 5.09 | 0.78 |
| 99 | 8 | 1623 | 0.108 | 2.3 | 0.81 | 7.52 | 0.35 |
| 100 | 8 | 1624 | 0.294 | 3.3 | 2.66 | 9.04 | 0.81 |
| 101 | 8 | 1625 | 0.21 | 2.7 | 1.59 | 7.57 | 0.59 |
| 102 | 8 | 1632 | 0.478 | 1.6 | 1.85 | 3.88 | 1.16 |
| 103 | 11 | 783 | 0.238 | 2.5 | 0.55 | 2.31 | 0.22 |
| 104 | 11 | 1563 | 0.112 | 2.6 | 2.43 | 21.71 | 0.94 |
| 105 | 11 | 1564 | 0.440 | 2.5 | 1.49 | 3.38 | 0.59 |
| 106 | 11 | 1595 | 0.241 | 1.9 | 1.55 | 6.44 | 0.82 |
| 107 | 11 | 1634 | 0.23 | 3.5 | 1.34 | 5.84 | 0.38 |
| 108 | 5 | 768 | 0.421 | 2.1 | 2.12 | 5.03 | 1.01 |
| 109 | 5 | 1536 | 0.107 | 2.4 | 1.28 | 12.00 | 0.54 |
| 110 | 5 | 1544 | 0.415 | 2.6 | 1.06 | 2.56 | 0.41 |
| 111 | 5 | 1569 | 0.279 | 2.2 | 5.06 | 18.13 | 2.30 |
| 112 | 5 | 1570 | 0.225 | 6.4 | 6.99 | 31.08 | 1.10 |
| 113 | 15 | 929 | 0.199 | 2.0 | 1.19 | 5.96 | 0.59 |
| 114 | 15 | 929 | 0.466 | 2.0 | 2.35 | 5.04 | 1.18 |
| 115 | 15 | 1604 | 0.225 | 2.7 | 4.44 | 19.74 | 1.64 |
| 116 | 15 | 1614 | 0.224 | 3.3 | 0.61 | 2.71 | 0.18 |
| 117 | 15 | 1614 | 0.287 | 2.4 | 1.28 | 4.47 | 0.53 |
| 118 | 6 | 1537 | 0.288 | 3.3 | 1.21 | 4.21 | 0.37 |
| 119 | 6 | 1550 | 0.269 | 3.2 | 2.86 | 10.65 | 0.89 |
| 120 | 6 | 1559 | 0.207 | 2.2 | 1.76 | 8.50 | 0.80 |
| 121 | 6 | 1560 | 0.234 | 2.3 | 1.25 | 5.33 | 0.54 |
| 122 | 9 | 467 | 0.269 | 2.4 | 4.10 | 15.24 | 1.71 |
| 123 | 9 | 918 | 0.269 | 3.8 | 0.86 | 3.21 | 0.23 |
| 124 | 9 | 1553 | 0.222 | 2.5 | 2.21 | 9.96 | 0.88 |
| 125 | 9 | 1628 | 0.217 | 2.2 | 2.12 | 9.77 | 0.96 |
| 126 | 11b | 461 | 0.078 | 3.4 | 0.46 | 5.84 | 0.13 |
| 127 | 11b | 461 | 0.25 | 2.9 | 0.63 | 2.54 | 0.22 |
| 128 | 11b | 1627 | 0.258 | 2.3 | 0.97 | 3.76 | 0.42 |
| 129 | 11b | 1627 | 0.214 | 2.3 | 1.09 | 5.07 | 0.47 |
| 130 | 10 | 1363 | 0.319 | 3.0 | 1.11 | 3.48 | 0.37 |
| 131 | 10 | 1631 | 0.279 | 3.4 | 1.35 | 4.85 | 0.40 |
| 132 | 14 | 1617 | 0.138 | 3.0 | 1.80 | 13.05 | 0.60 |
| 133 | 16 | 1545 | 0.495 | 1.8 | 0.79 | 1.59 | 0.44 |
| 134 | Singleton | 1538 | 0.148 | 2.6 | 1.11 | 7.50 | 0.43 |
| 135 | Singleton | 1538 | 0.273 | 2.8 | 2.50 | 9.15 | 0.89 |
| 136 | Singleton | 1540 | 0.263 | 2.4 | 3.35 | 12.76 | 1.40 |
| 137 | Singleton | 1541 | 0.21 | 2.5 | 3.00 | 14.27 | 1.20 |
| 138 | Singleton | 1543 | 0.277 | 3.0 | 2.69 | 9.71 | 0.90 |
| 139 | Singleton | 1548 | 0.253 | 2.7 | 0.52 | 2.04 | 0.19 |
| 140 | Singleton | 1549 | 0.25 | 3.5 | 0.78 | 3.13 | 0.22 |
| 141 | Singleton | 1552 | 0.138 | 2.8 | 2.75 | 19.90 | 0.98 |
| 142 | Singleton | 1554 | 0.282 | 3.3 | 1.50 | 5.33 | 0.46 |
| 143 | Singleton | 1558 | 0.28 | 3.2 | 0.97 | 3.47 | 0.30 |
| 144 | Singleton | 1561 | 0.276 | 1.5 | 0.53 | 1.93 | 0.35 |
| 145 | Singleton | 1575 | 0.322 | 3.2 | 2.83 | 8.79 | 0.88 |
| 146 | Singleton | 1575 | 0.24 | 2.3 | 0.98 | 4.08 | 0.43 |
| 147 | Singleton | 1620 | 0.222 | 2.8 | 0.61 | 2.75 | 0.22 |
| 148 | Singleton | 1633 | 0.279 | 2.7 | 1.16 | 4.17 | 0.43 |
| 149 | Singleton | 1635 | 0.305 | 2.7 | 1.24 | 4.05 | 0.46 |
|  | | | | | | | |

^a^ For each isolate, the FOH level in 24-hour biofilms was determined once in filtered (0.45 μm) culture supernatant using high-performance liquid chromatography. The excellent reproducibility (CV <5%) of FOH results by HPLC was obtained using five samples tested three times on three separate days.

^b^ The value were normalized on a metabolic basis by dividing the FOH values (μM) by the biofilm results determined by XTT reduction (OD_492_).

^c^ The value were normalized on a per-weight basis by dividing the FOH values (μM) by dry weight (mg) assays, respectively.
